# The effects of combination-therapy of tocilizumab and baricitinib on the management of severe COVID-19 cases: a randomized open-label clinical trial

**DOI:** 10.3389/fphar.2023.1265541

**Published:** 2023-10-19

**Authors:** Farzaneh Dastan, Hamidreza Jamaati, Saghar Barati, Shahrzad Varmazyar, Sahar Yousefian, Elmira Niknami, Payam Tabarsi

**Affiliations:** ^1^ Department of Clinical Pharmacy, School of Pharmacy, Shahid Beheshti University of Medical Sciences, Tehran, Iran; ^2^ Chronic Respiratory Diseases Research Center, National Research Institute of Tuberculosis and Lung Diseases (NRITLD), Shahid Beheshti University of Medical Sciences, Tehran, Iran; ^3^ Clinical Tuberculosis and Epidemiology Research Centre, National Research Institute for Tuberculosis and Lung Disease (NRITLD), Shahid Beheshti University of Medical Sciences, Tehran, Iran

**Keywords:** baricitinib, COVID-19, inflammation, pneumonia, SARS-CoV-2, tocilizumab

## Abstract

**Background:** Tocilizumab and baricitinib are considered standard treatments for hospitalized COVID-19 patients with an inflammatory status. However, the effects of co-administering these medications aiming for more rapid patient recovery are controversial among practitioners. The potential benefits include the rapid improvement of patients and regulation of the immune system, and the potential risks include the increased chance of serious adverse events, including infections. This study aimed to investigate the effects of co-administering these two medications on the 28-day mortality rate, other efficacy parameters, and safety issues.

**Methods:** In this randomized open-label trial, 68 patients were recruited. The study was conducted at Dr. Masih Daneshvari Hospital during 6 months (from 21 March 2022 to 23 August 2022). Severely ill patients aged between 18 and 100 years old with confirmed COVID-19 were enrolled. The primary outcomes included the need for invasive mechanical ventilation and a 28-day mortality rate. Secondary outcomes included the need for non-invasive mechanical ventilation, the need for admission to the intensive care unit (ICU), the length of hospital stay, and the need for a second dose of tocilizumab. Safety assessments were also performed for 28 days. The data were collected from the patients’ medical records, which included age, gender, and comorbidities.

**Results:** The 28-day mortality rate or the need for mechanical ventilation was not statistically different among the two groups (*p*-value = 0.49 for both outcomes). The need for non-invasive mechanical ventilation, the need for admission to the ICU, or the need for a second dose of tocilizumab and the length of hospital stay was not affected either (*p*-value = 1; 0.1; 0.49 and 0.9, respectively). One patient developed thrombosis in the combination group. No adverse events related to infectious complications were recorded in any groups.

**Conclusion:** This study showed no beneficial effects of combining tocilizumab and baricitinib in managing severe COVID-19 cases. However, the need for ICU admission was meaningfully lower in the combination group. Studies with larger sample sizes are needed to confirm these results.

**Clinical Trial Registration:** Identifier: RCT20151227025726N30M

## 1 Introduction

The coronavirus disease 2019 (COVID-19) pandemic, caused by the novel severe acute respiratory syndrome coronavirus 2 (SARS-CoV-2), has had a profound and lasting impact on global health and society. Since its emergence in Wuhan, China, in late 2019, COVID-19 has spread relentlessly across the globe, challenging healthcare systems, economies, and daily life as we know it. As of September 2023, the global rate of SARS-CoV-2 infections remains high, with seroprevalence studies showing that> 80% of the population of countries such as South Africa have already been infected (Hajissa et al., 2022). The treatment strategies for COVID-19 depend on its stage and should be tailored to the specific stage of the disease that a patient is experiencing (Stasi et al., 2020; Wang et al., 2023).

Inflammatory markers play a crucial role in the pathogenesis of COVID-19, contributing to the disease’s severity and complications. One of the hallmark features of severe COVID-19 is a hyperinflammatory response often referred to as a “cytokine storm” (Mehta et al., 2020). This involves the overproduction of pro-inflammatory cytokines, including interleukin-6 (IL-6), interleukin-1 beta (IL-1β), and tumor necrosis factor-alpha (TNF-α). This excessive cytokine release can lead to acute respiratory distress syndrome (ARDS) and multi-organ dysfunction (Dastan et al., 2020; Mehta et al., 2020; Rezaei et al., 2021; Rubin et al., 2021). Until now, many antiviral drugs and monoclonal antibodies (bebtelovimab, sotrovimab and etc.) have been used for the treatment of COVID-19. One of the most promising drugs is remdesivir which is an adenosine analogue and has antiviral activity (Li et al., 2023; Mengato et al., 2023). Tocilizumab is an interleukin-6 (IL-6) receptor blocker currently approved for hospitalized patients with high inflammatory status (Samaee et al., 2020). It promotes B and T cells' proliferation, which may trigger further immune activation (Uciechowski and Dempke, 2020). COVID-19 patients treated with tocilizumab have a lower mortality rate and a lower need for mechanical ventilation (Wei et al., 2021). Other interleukin blockers including anakinra which inhibit interleukin-1 have been used in the management of COVID-19 before. Some studies have suggested potential benefits of this medication, including improved oxygenation and reduced need for mechanical ventilation, in patients with severe COVID-19 and signs of hyperinflammation (Cavalli et al., 2020).

The Janus kinase (JAK) inhibitor, baricitinib, inhibits the passage and intracellular assembly of SARS-CoV-2 into target cells through the ACE2 receptor (Keystone et al., 2015). Moreover, this medication suppresses both JAK1/JAK2, which leads to inhibiting the proinflammatory signal of several cytokines, including IL-6, IL-12, IL-23, and IFN-γ (Keystone et al., 2015).

The indication for tocilizumab or baricitinib is almost similar in COVID-19 patients. Patients with a rapid disease progression, high oxygen needs on admission, or patients with risk factors for poor outcomes, including age or medical history plus elevated C-reactive protein (CRP) plus low-flow oxygen, did receive tocilizumab or baricitinib (Karolyi et al., 2022). Unlike tocilizumab, baricitinib could be prescribed at any CRP level, while tocilizumab required a CRP of 75 mg/L (Karolyi et al., 2022).

The effects and outcomes of co-administering these two medications have yet to be assessed in well-designed trials. The potential benefits include the rapid improvement of patients and regulation of the immune system. The potential risks include the increased chance of infections due to over-suppression of the immune system and further immune issues. However, the short duration of administration may reduce the importance of this concern. This study aimed to investigate the effects of co-administering these two medications on the 28-day mortality rate, the need for mechanical ventilation, along with other efficacy parameters and safety issues.

## 2 Materials and methods

### 2.1 Setting

This study was a randomized, open-labeled, parallel-group, two-armed, single-center clinical trial conducted on 68 COVID-19 patients at Dr. Masih Daneshvari Hospital, affiliated with Shahid Beheshti University of Medical Sciences (SBMU), a referral centre for COVID-19 patients in Tehran, Iran. A block randomization method was used to allocate patients to the tocilizumab group and tocilizumab + baricitinib group.

### 2.2 Patients

Severely ill patients aged between 18 and 100 years old with COVID-19, confirmed based on the reports of Reverse Transcription-Polymerase Chain Reaction (RT-PCR) and who have also signed the study consent form, were allowed to be recruited. Severely ill patients were defined as any of the following: respiratory rate of 30 or more breaths per minute; heart rate at or exceeding 125 beats per minute; oxygen saturation at 93% or less while the participant was breathing ambient air at sea; acute respiratory distress syndrome and evidence of shock (Baden et al., 2021).

The exclusion criteria were as follows: patients who denied signing the consent form; an acute or chronic kidney disease defined as a rise in serum creatinine greater than 0.3 mg/dL within 48 h or a lower glomerular filtration rate than 30 mL/min; the history of liver failure (Child-Pugh stage C or D or more than five times the upper limit of normal in liver function tests or three times in patients with liver failure symptoms); patients with a history of an allergic reaction to the study medications or any history of anaphylaxis to any medication; mildly ill patients; patients needing mechanical ventilation at admission, and pregnancy or breastfeeding.

### 2.3 Interventions

Patients were randomized to two equal sample sizes. The intervention group received a single intravenous dose of 400 mg tocilizumab (Temziva^®^, AryoGen Pharmed, Iran) via slow intravenous infusion plus 4 mg baricitinib (Intyma^®^, Nanoalvand, Iran) daily for 14 days or until discharge. The control group received a single intravenous dose of 400 mg tocilizumab (Temziva^®^, AryoGen Pharmed, Iran) via slow intravenous infusion. Patients in both groups received oxygen and fluid support, remdesivir 200 mg stat followed by 100 mg daily intravenously for 5 days, and dexamethasone 6 mg intravenously daily for 10 days or until discharge. The concomitant medications were the same in the both groups.

### 2.4 Outcomes

The primary outcomes included the need for invasive mechanical ventilation and the 28-day mortality rate. Secondary outcomes included the need for non-invasive mechanical ventilation, the need for admission to the intensive care unit (ICU), the length of hospital stay, and the need for second dose of tocilizumab. The data were collected from the patients’ medical records, which included age, gender, underlying diseases, and laboratory test results.

Safety assessments were performed for 28 days after the first day of the intervention. These statements were made based on physicians’ reports in the hospital. After discharge, safety assessments were performed based on follow-up telephone calls, and these assessments were patient-reported.

### 2.5 Statistical analysis

Overall, 68 participants were enrolled in the study. This sample size was not determined based on any power calculation and was chosen according to the available COVID-19 patients needing tocilizumab at admission.

Missing data were not imputed. No multiplicity adjustments were made in this study. Based on the central limit theorem, the normality tests were not performed, as this theorem believes that the sampling distribution of the mean will always be normally distributed, as long as the sample size is large enough (Kwak and Kim, 2017).

Categorical variables were expressed as frequency (%) and were compared using the Chi-Square or Fisher exact test. The risk difference was calculated as a proper effect size for the primary outcome. Continuous variables were expressed as mean ± standard deviations. To compare the differences in the quantitative variables of both groups, the student-t test was carried out. *p*-values <0.05 were considered significant.

All the statistical analyses were performed using SPSS software for Windows (Version 23.0; SPSS Inc., Chicago, IL, United States) and STATA 14.

### 2.6 Ethical considerations

This trial was conducted according to the declaration of Helsinki and was approved by the ethics committee of Shahid Beheshti University of Medical Sciences (IR.SBMU.PHARMACY.REC.1400.296). Written informed consent was obtained from all the participants before recruitment.

Moreover, the trial was registered in Iranian Registry of Clinical Trials with the registration code IRCT20151227025726N30.

## 3 Results

**FIGURE 1:**
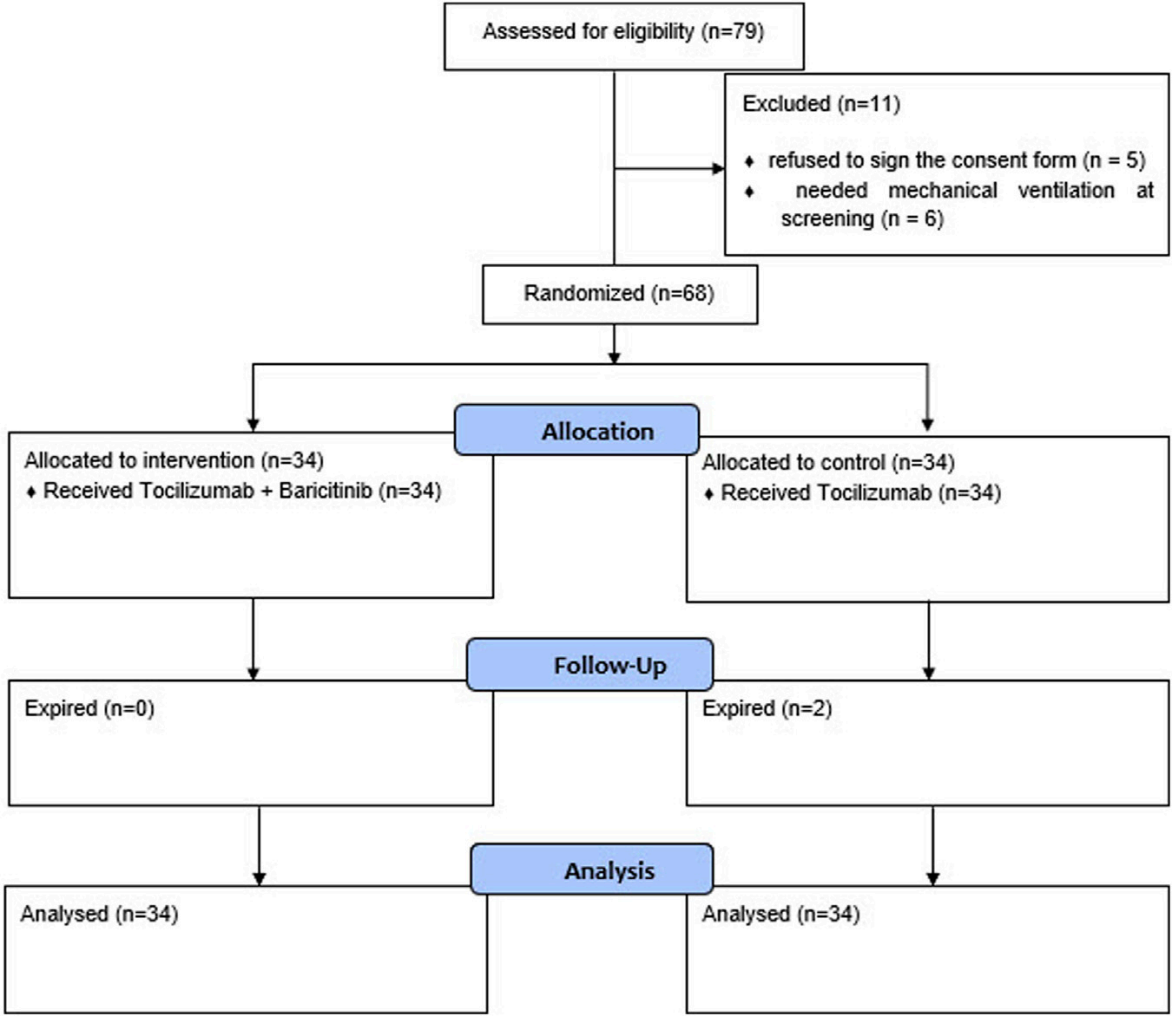
Screening and randomization process of the patients.

The screening and randomization process of the patients are provided in the CONSORT diagram in Figure 1.

Table 1 demonstrates the demographics and past medical histories of the subjects.

**TABLE 1:** Demographics and baseline characteristics of the patients.

Characteristics	Tocilizumab (N = 34)	Tocilizumab + baricitinib (N = 34)	*p*-value
Sex–n (%) Male	24 (70.58)	22 (64.7)	0.6
Age (year)–mean ± SD	61.32 ± 17.1	69.21 ± 16.62	0.058
Smoking (yes)	6 (17.64)	2 (5.88)	0.25
Asthma (yes)	1 (2.94)	1 (2.94)	1
Hypertension (yes)	15 (44.11)	17 (50)	0.62
Diabetes mellitus (yes)	8 (23.52)	9 (26.47)	0.77
Chronic kidney diseases (yes)	3 (8.82)	1 (2.94)	0.61
Chronic obstructive pulmonary disease (yes)	2 (5.88)	0 (0)	0.49
Rheumatoid arthritis (yes)	1 (2.94)	0 (0)	1
Malignancy (yes)	5 (14.7)	4 (11.76)	1

The results regarding the 28-day mortality rate, the need for invasive or non-invasive mechanical ventilation, and the need for the second tocilizumab dose or admission in the ICU are provided in Table 2. As the Table shows, there were no statistical differences in terms of the mentioned parameters between the two groups (Table 2).

**TABLE 2:** Results of the study outcomes.

Outcomes	Tocilizumab (N = 34)	Tocilizumab + baricitinib (N = 34)	*p*-value
28-day mortality rate	2 (5.88%)	0 (0%)	0.18
The need for mechanical ventilation	2 (5.88%)	0 (0%)	0.18
The need for non-invasive mechanical ventilation	5 (14.7%)	4 (11.76%)	1
The need for tocilizumab second dose	10 (29.41%)	10 (29.41%)	0.42
The need for admission to the ICU	6 (17.64%)	1 (2.94%)	0.1
Length of hospital stay (Day)	10.55 (6.34)	10.41 (5.37)	0.9

Data are presented as n (%); ICU, intensive care unit.

### 3.1 Safety outcomes

The lab tests regarding the assessments of the patients' inflammatory status and safety assessments are provided in Table 3.

**TABLE 3:** Comparison of the lab tests between the two groups.

Lab tests	Tocilizumab	Total	Tocilizumab + baricitinib	Total	*p*-value
Cr before Intervention (mg/dL)	1.51 ± 0.49	21	1.33 ± 0.36	24	0.18
Cr 5 days after Intervention (mg/dL)	1.44 ± 0.35	17	1.24 ± 0.33	20	0.09
AST before Intervention (IU/L)	44.72 ± 34.82	18	45.7 ± 25.81	17	0.92
AST 5 days after Intervention (IU/L)	41 ± 22.42	15	33.46 ± 12.92	13	0.29
ALT before Intervention (IU/L)	45 ± 48.32	18	46.47 ± 45.32	17	0.92
ALT 5 days after Intervention (IU/L)	81 ± 99.02	15	41.07 ± 23.35	13	0.15
CRP before Intervention (mg/dL)	46.26 ± 27.09	17	56.34 ± 19.63	20	0.21
CRP 5 days after Intervention (mg/dL)	12.61 ± 17.61	11	14.4 ± 11.15	14	0.75
D-Dimer before Intervention (mg/L)	1,197.44 ± 1,060.95	34	771.5 ± 905.43	30	0.09
D-Dimer 3 days after Intervention (mg/L)	1,255.77 ± 12.08.03	27	1,034.86 ± 1,105.24	23	0.5

Data are presented as Mean ± SD; Cr, creatinine; AST, aspartate aminotransferase; ALT, alanine aminotransferase; CRP, C-Reactive protein; IL-6, Interleukin-6.

One patient in the tocilizumab + baricitinib group developed thrombosis. No other adverse events related to the study treatments were reported.

## 4 Discussion

The results of this study showed no beneficial effects of combining tocilizumab and baricitinib on the management of severe COVID-19 cases. The health outcomes, including the need for mechanical or non-invasive mechanical ventilation, and the mortality rate were not different between the groups receiving the combination of these two medications and the group receiving a single dose of tocilizumab. The days of hospital stay or the need for second dose of tocilizumab was not affected either.

It is noteworthy that the risk difference in the need for admission to the ICU is calculated to be 0.15. Based on this calculation, the number need to treat (NNT) to prevent one person from admitting to the ICU is 7, which is considered a medium effect size (Rahlfs and Zimmermann, 2019). Hence, combination therapy may have beneficial effects on the prevention from ICU admission. However, the sample size needed to be larger to detect that beneficial effect.

Baricitinib became the first immunomodulatory treatment for COVID-19 to receive FDA approval. This approval was supported by two phase 3, randomized, double-blind placebo-controlled trials (Rubin, 2022). Based on a review article, baricitinib was effective in decreasing respiratory failure and the use of mechanical ventilation, also preventing symptoms deterioration. Furthermore, baricitinib as a single agent or combined with other drugs, improved the peripheral capillary oxygen saturation (SpO2)/fraction of inspired oxygen (FiO2) ratio (Dupuis et al., 2022).

Both baricitinib and tocilizumab are considered a standard of care in managing severe COVID-19 patients with inflammatory status based on COVID-19 treatment guidelines (Bhimraj et al., 2020). Some other studies have also considered the co-administration of these two medications, which may lead to more rapid recovery of the patients from the inflammatory status as they both suppress the immune system (Rosas et al., 2020). The authors of that study believed that the combination therapy of these medications could be considered in COVID-19 patients with impaired PaO_2_/PaFi. No serious adverse events were reported in their study (Rosas et al., 2020). In one study, authors concluded that using baricitinib along with standard of care treatments was associated with mortality reduction in hospitalized COVID-19 patients (Banga et al., 2023).

Both tocilizumab and baricitinib have anti-inflammatory properties. Combining these medications may result in a more comprehensive suppression of the hyperinflammatory response often seen in severe COVID-19 cases (Bryus et al., 2022).

In our study, one patient developed thrombosis. The relation of this event to the study intervention may not be conclusive as COVID-19 is a risk factor for developing thrombotic events itself. However, it should be noted that the use of baricitinib also may increase this risk. No infectious-related complications or any issues related to over-suppression of the immune system were noted.

In another observational cohort study performed on microbiologically-confirmed COVID-19 hospitalizations, the addition of baricitinib to the standard of care, including tocilizumab did not reduce the mortality rate in hospitalized COVID-19 patients, which is in line with our results (Masiá et al., 2021). Moreover, no difference in the thromboembolic events or infection rates was detected in that study (Masiá et al., 2021). While some studies have shown promise, the evidence regarding the combined use of tocilizumab and baricitinib for COVID-19 is still evolving. More research is needed to establish the safety and efficacy of this combination definitively. It is noteworthy that both tocilizumab and baricitinib are expensive medications, and using them together may significantly increase the overall treatment cost. The decision to combine these medications should be made on a case-by-case basis, taking into account the patient’s overall health, comorbidities, and individual response to treatment.

The results of the lab tests, demonstrates no significant and meaningful differences regarding the safety issues or the inflammation reduction between the two groups.

While some studies have shown promise, the evidence regarding the combined use of tocilizumab and baricitinib for COVID-19 is still evolving. More research is needed to establish the safety and efficacy of this combination definitively.

This study had some limitations. The main limitation of our study was the absence of a power calculation for sample size determination. Nevertheless, given the calculated risk difference of 0.05 in the primary outcomes, which represents a trivial effect size, it appears that increasing the sample size would not alter the results. In addition, the open-label design of the study could have affected the results which must be taken into account.

Moreover, according to the participants’ baseline characteristics, the patients’ smoking status might be considered a covariate in this study. However, the low number of the events related to the primary outcomes was a limitation for performing adjustment analysis based on regression models.

## 5 Conclusion

The combination therapy of tocilizumab plus baricitinib did not have any beneficial effects on managing severe COVID-19 cases. However, the need for ICU admission was meaningfully lower in the combination group.

## Data Availability

The raw data supporting the conclusion of this article will be made available upon a reasonable request to the corresponding author.
